# Rapamycin prevents lung injury related to acute spinal cord injury in rats

**DOI:** 10.1038/s41598-023-37884-6

**Published:** 2023-07-01

**Authors:** Ruiliang Chu, Nan Wang, Yang Bi, Guoxin Nan

**Affiliations:** 1 Department of Orthopedics Children’s Hospital of Chongqing Medical University, National Clinical Research Center for Child Health and Disorders, Ministry of Education Key Laboratory of Child Development and Disorders, China International Science and Technology Cooperation Base of Child Development and Critical Disorders, Chongqing Engineering Research Center of Stem Cell Therapy, Chongqing, China; 2 Present Address: Dongguan Children’s Hospital Affiliated to Guangdong Medical University, Dongguan Eighth People’s Hospital, Dongguan Institute of Pediatrics, Dongguan, China

**Keywords:** Autophagy, Comorbidities, Experimental models of disease, Trauma

## Abstract

Severe injury occurs in the lung after acute spinal cord injury (ASCI) and autophagy is inhibited. However, rapamycin-activated autophagy's role and mechanism in lung injury development after ASCI is unknown. Preventing lung injury after ASCI by regulating autophagy is currently a valuable and unknown area. Herein, we aimed to investigate the effect and possible mechanism of rapamycin-activated autophagy on lung damage post-ASCI. An experimental animal study of rapamycin's effect and mechanism on lung damage after ASCI. We randomly divided 144 female wild-type Sprague–Dawley rats into a vehicle sham group (n = 36), a vehicle injury group (n = 36), a rapamycin sham group (n = 36), and a rapamycin injury group (n = 36). The spine was injured at the tenth thoracic vertebra using Allen's method. At 12, 24, 48, and 72 h after surgery, the rats were killed humanely. Lung damage was evaluated via pulmonary gross anatomy, lung pathology, and apoptosis assessment. Autophagy induction was assessed according to LC3, RAB7, and Beclin 1 levels. ULK-1, ULK-1 Ser555, ULK-1 Ser757, AMPK α and AMPK β1/2 were used to investigate the potential mechanism. After rapamycin pretreatment, the lung showed no obvious damage (e.g., cell death, inflammatory exudation, hemorrhage, and pulmonary congestion) at 12 h and 48 h after injury and Beclin1, LC3 and RAB7 levels increased. After rapamycin pretreatment, ULK-1, ULK-1 Ser555, and ULK-1 Ser757 levels increased at 12 h and 48 h after injury compared with the vehicle group, but they decreased at 12 h after injury compared with the rapamycin sham group. After rapamycin pretreatment, AMPKα levels did not change significantly before and after injury; however, at 48 h after injury, its level was elevated significantly compared with that in the vehicle group. Rapamycin can prevent lung injury after ASCI, possibly via upregulation of autophagy through the AMPK–mTORC1–ULK1 regulatory axis.

## Introduction

Acute spinal cord injury (ASCI) is one of the most devastating traumatic injuries. The damage caused by ASCI not only includes permanent neurological damage, but also non-neurological complications that directly increase the mortality of patients^1^. DeVivo et al.'s research showed that the highest mortality factor after ASCI is respiratory system disease, and reducing respiratory system complications is the key to preventing the death of patients; however, despite advances in medical technology, there is still no effective way to prevent lung injury after ASCI^2^.

An early study reported that respiratory muscle paralysis caused by nerve injury was the main cause of respiratory dysfunction and decreased respiratory clearance^3^. However, the nerves that innervate the respiratory muscles are located above the upper thoracic region and our previous research showed that low (T10) ASCI animal models (in which the location of the spinal cord injury avoids the upper thoracic region that innervates the respiratory muscles) still experienced lung injuries, such as pulmonary hemorrhage, edema, and inflammatory infiltration^4^. Analysis of a large amount of clinical data revealed that in patients with spinal cord injury, regardless of whether the injury site occurred in the cervical spine, thoracic spine, or lumbar spine, the incidence of lung injury is the highest among internal organs (more than a quarter)^5^. Thus, respiratory muscle paralysis cannot explain the development of lung injury post-ASCI. The most frequent respiratory complication after ASCI that causes death is pneumonia (65.1%), accompanied by sepsis. However, there were a large number of cases (48.5%) in which infection occurred without a clear source^2^. This situation leads to the inability to reverse lung inflammation via an anti-infection treatment plan after using antibiotics in clinical treatment, and eventually leads to death. This shows that controlling infection alone cannot prevent lung injury post-ASCI. The role of autophagy in acute lung injury caused by different factors is inconsistent, while the role of autophagy in ASCI-induced lung injury is still unknown.

Previously, our research showed that autophagy was inhibited in the lung after ASCI^6^, which suggests a new target to treat lung injury post-ASCI. Rapamycin can activate autophagy by specifically inhibiting mTOR. Accordingly, the present study used rapamycin to upregulate autophagy and observe its effect on lung injury following ASCI, and preliminarily investigated its possible mechanism, providing a new theoretical basis to prevent the occurrence of lung injury post-ASCI.

## Materials and methods

### Animals

We used 144 female wild-type Sprague–Dawley rats (weight: 200–220 g), which were randomly divided into a vehicle (veh) sham group (n = 36), a vehicle injury group (n = 36), a rapamycin (rapa) sham group (n = 36), and rapamycin injury group (n = 36). The rats were assigned to the groups using a random number table. At 12, 24, 48, and 72 h after treatment, nine rats in each group were killed humanely. The animals were reared at room temperature (22 to 26 °C) at 40–60% relative humidity and a light/dark cycle of 12/12 h. In the rapamycin groups, rapamycin (1 mg/kg^7^ of body weight; Selleck Chemicals, Houston, TX, USA) was administered intraperitoneally at 15 min before ASCI. In the vehicle group, the rats received the same volume of vehicle (0.9% normal saline, 5% Tween-80, 5% polyethylene glycol-400; Beyotime, Shanghai, China) intraperitoneally. All experimental protocols were approved by the Association for Assessment and Accreditation of Laboratory Animal Care International, China and the Experimental Animal Committee of Chongqing Medical University accredited all the animal studies (Permit numbers: SCXK [Yu] 2022–0010 and SYXK [Yu] 2022–0016). All methods were reported in accordance with ARRIVE guidelines. During all procedures, pain was minimized using the anesthetic sodium pentobarbital. Rats were euthanized by injection of anaerobic sodium pentobarbital.

### Model of acute spinal cord injury

In the experiment, we used the method developed by Allen to induce injury to the rat thoracic spinal cord^6^. The rats were injected with sodium pentobarbital at 10 g/L (40 mg/kg, Sigma-Aldrich, St. Louis, MO, USA) for anesthesia. The rats were then fixed onto the operating table, where their skin was prepared and sterilized. An incision was made dorso-thoracically from T9 to T11 to expose the erector spinae. T10 laminectomy (removal of the T10 lamina) was carried out to expose the spinal cord, which was then covered with a thin copper slice. We dropped a 10 g metal bar from a height of 2.5 cm through a graduated glass tube onto the copper slice, after which the copper covering was quickly removed. Rat tail swinging or curling indicated successful injury. In the control group, the rats underwent a T10 laminectomy only. Post-surgery, the incision site was closed using layer by layer sutures, and the rats were allowed to recover in an air-conditioned room. Their bladders were emptied manually three times daily.

### Pulmonary edema evaluation

Pulmonary edema was indicated using the wet-to-dry weight (W/D) ratio. At various time points post-surgery or sham surgery, the rats were sacrificed, their lungs were excised and separated into the left lung and the anterior, middle, posterior, and accessory lobes of the right lung. The wet weights of the lung samples were obtained immediately, and after 72 h of drying at 70 °C, the dry weights were obtained.

### Histological analysis of rat lung tissue

Normal saline (40 mL) and 4% paraformaldehyde (20 mL) were instilled into the lungs. After excision from the thoracic cavity, fixation of the lungs was achieved using 4% paraformaldehyde for 48 h, followed by paraffin embedding, sectioning at 4 μm sections, and staining with hematoxylin and eosin (HE) for light microscopy evaluation. For lung sections from the sham injury groups, in situ detection of fragmented DNA was conducted using TdT-mediated dUTP-nick end labeling (TUNEL) fluorescent analysis, following the supplier's guidelines (KeyGEN BioTECH; Jiangsu, China). Briefly, deparaffinization of the paraffin sections was followed by rehydration and proteinase K digestion for 30 min at 37 °C. The sections were rinsed with phosphate buffered saline (PBS) for 15 min, and then incubated with biotin-11-dUTP and TdT enzyme in the dark for 60 min at 37 °C. The sections were washed and then incubated in streptavidin-fluorescein for 30 min at 37 °C in the dark. Finally, the cell nuclei were stained using 4′,6-diamidino-2-phenylindole (DAPI)(Sigma-Aldrich), and the sections were viewed under a fluorescence microscope. Image analysis was performed using ImageJ 1.40 g software (NIH, Bethesda, MD, USA), and TUNEL-positive cells were counted blindly in 10 randomly selected fields at 200 × magnification.

### Western blotting analysis

We homogenized lung tissues in lysis buffer (20 mM Tris (pH 7.5), 1.0 mM Phenylmethanesulfonyl fluoride,1% Triton X-100, and 150 mM NaCl). The tissue lysates were centrifuged at 4 °C for 5 min at 13,000 × g. The resulting total proteins were mixed with 5 × SDS-PAGE loading buffer and subjected to electrophoresis on Tris–glycine gels. The separated proteins were then transferred electrophoretically onto polyvinylidene fluoride membranes. 10% skim milk was then used to block the membranes for 1 h at 37 °C. Next, the membranes were incubated at 4 °C overnight with antibodies recognizing Beclin 1 (Abcam, Cambridge, MA, USA), microtubule associated protein 1 light chain 3 alpha (LC3) (Cell Signaling Technology, Danvers, MA, USA), Ras-associated protein RAB7 (Abcam), Unc-51 like autophagy activating kinase 1 (ULK-1) (Cell Signaling Technology), ULK-1 Ser555 (Cell Signaling Technology), ULK-1 Ser757 (Cell Signaling Technology), AMP-activated protein kinase, catalytic, alpha-1 subunit (AMPKα) (Cell Signaling Technology), and AMP-activated protein kinase, catalytic, beta-1/2 subunits (AMPKβ1/2) (Cell Signaling Technology). Thereafter, TBST (Tris–HCl 8.0, NaCl, 0.1% Tween 20) was used to wash the membranes, followed by incubation with horseradish-peroxidase labeled secondary antibodies. After a final wash, the immunoreactive proteins were detected using enhanced chemiluminescence (Bio-Rad, Hercules, CA, USA). The loading control was beta actin.

### Immunofluorescent Staining

Lung tissue paraffin Sects. (4 μm thick) were dewaxed and hydrated. Antigen retrieval was then performed by microwaving the sections in sodium citrate buffer (pH 6.0) for 15 min and cooling to room temperature. Then, 0.3% hydrogen peroxide was used to block endogenous peroxidase activity in the sections, followed by PBS washing for 15 min. Subsequently, 5% goat serum was used to block the samples for 1 h at room temperature. After discarding the goat serum, the samples were incubated overnight at 4 °C with anti-LC3 antibodies (Cell Signaling Technology) and anti-RAB7 antibodies (Sigma-Aldrich). After being returned to room temperature, the sections were PBS-washed. Alexa Fluor 488-conjugated goat anti-rabbit IgG H&L (Abcam) and Alexa Fluor 555-conjugated donkey Anti-Mouse IgG H&L (Abcam) were then incubated with a portion of the sections, followed by PBS washing, DAPI staining, and observation under a laser confocal microscope (Nikon A1R, Tokyo, Japan).

### Statistical considerations

Data are shown as the mean ± the standard error of the mean (SEM). To compare the groups at each time point, one-way analysis of variance (ANOVA) was used, followed by a post-hoc pairwise multiple comparison between the injured and sham groups at each post-instillation time point (12, 24, 48, and 72 h) using the least significant difference (LSD) method. Statistical significance was accepted at a *P* value < 0.05. SPSS 22.0 software (IBM Corp., Armonk, NY, USA) was used for all the statistical analyses.

### Ethics statement

All methods were performed in accordance with the relevant guidelines and regulations.

## Results

### Gross pulmonary anatomy after ASCI

**Figure 1:**
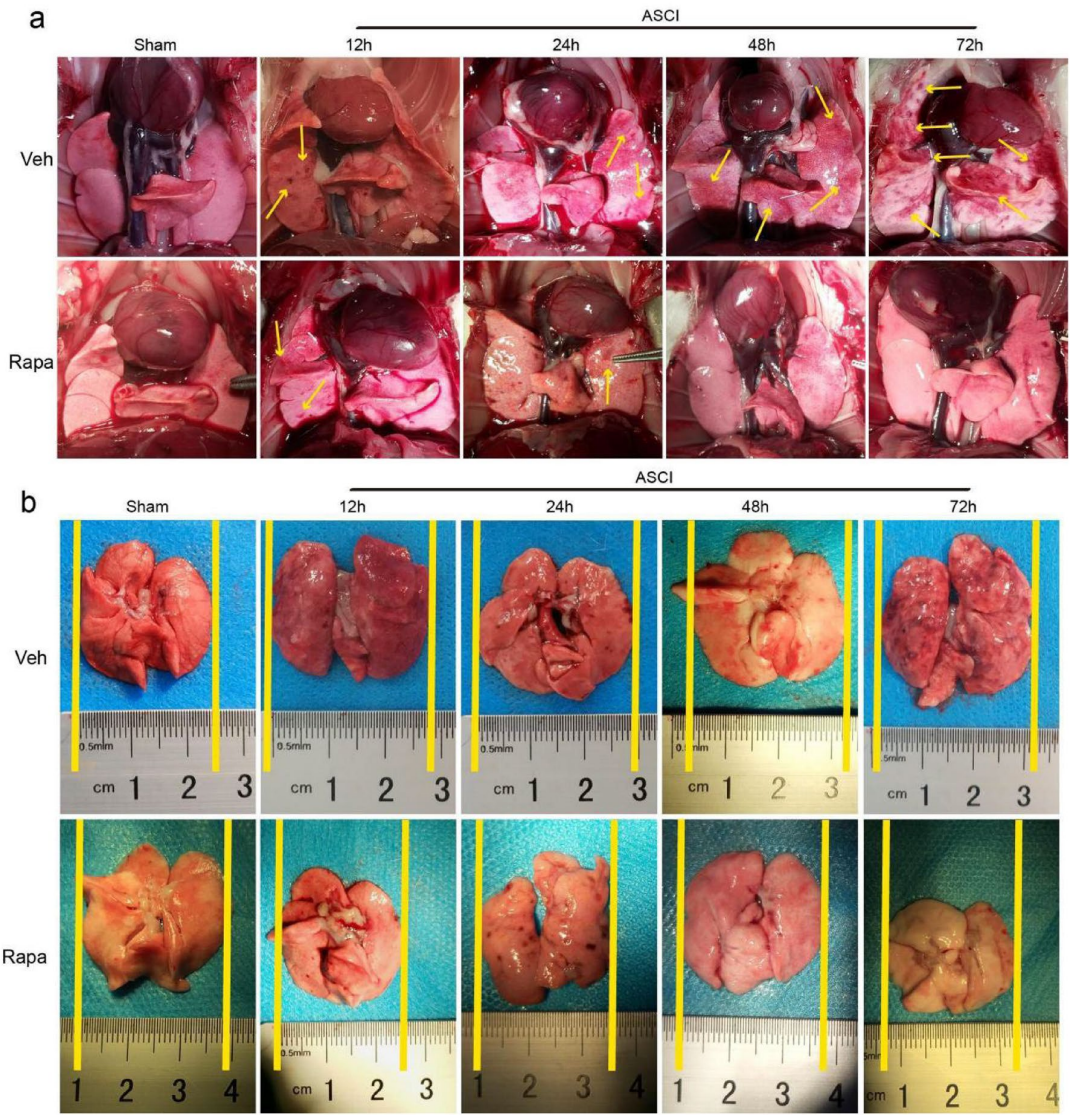
Pulmonary gross anatomy at different time points after Veh and Rapa pre-treatment and sham surgery or injury. (**a**) The state before infusion and blood removal without using physiological saline. The yellow arrow indicates the site of hemorrhage. (**b**) The state after infusion and blood removal by heart–lung perfusion using physiological saline. Veh, vehicle; Rapa, rapamycin; ASCI, acute spinal cord injury.

Rats were anesthetized with sodium pentobarbital and sacrificed at different time points to observe the gross pulmonary anatomy. The state before infusion and blood removal without using physiological saline is shown in Fig. 1a. The Veh group showed bleeding in the lung tissue after ASCI (yellow arrow) and the bleeding sites gradually increased over time. The Rapa group showed only a few bleeding points within 24 h, which did not increase over time and eventually returned to normal. The state after infusion and blood removal by heart–lung perfusion using physiological saline is shown in Fig. 1b. The Veh group showed an increase in lung tissue volume over time after ASCI. The Rapa group showed no significant change in lung tissue volume.

### Post-ASCI lung injury

**Figure 2:**
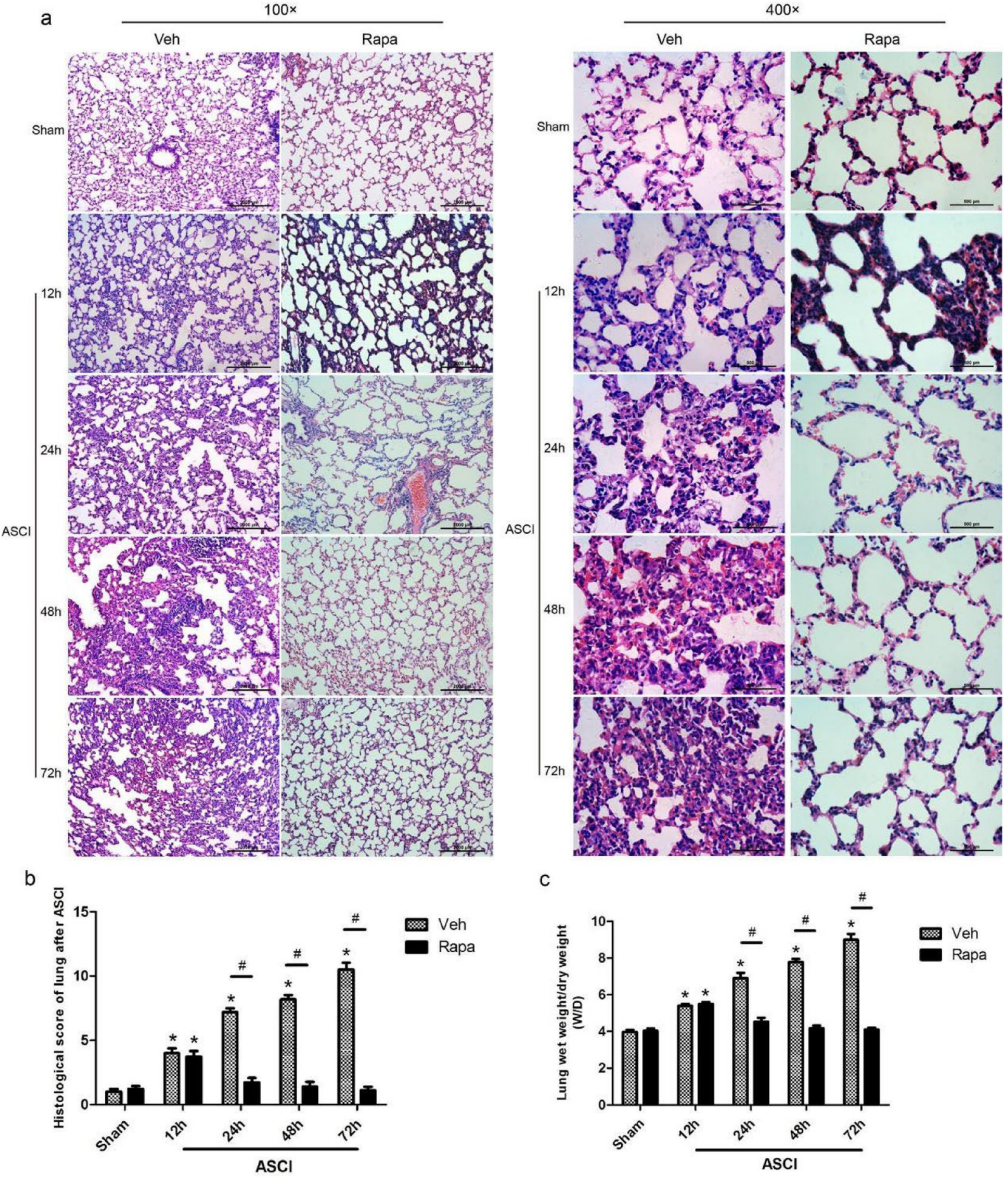
Evaluation of the damage/injury status of the lungs in each group at different time points. (**a**) Hematoxylin and eosin-stained sections of lung tissue showing alveolar stroma thickening, inflammatory exudation, hemorrhage, and pulmonary congestion. Representative results at 12, 24, 48 and 72 h are shown. Original magnification × 100 and × 400. (**b**) Pathological scoring of lung tissue sections (mean ± SD). (**c**) Lung wet-to-dry ratios at 12, 24, 48 and 72 h (mean ± SD). One-way analysis of variance and the Least Significant Difference test were used to compare the data. *P < 0.05 vs. the sham group (#*p* < 0.05). Veh, vehicle; Rapa, rapamycin; ASCI, acute spinal cord injury.

At various time points, H&E stained lung tissue slices were observed histologically (Fig. 2a). Compared with the Veh sham group, the Veh injury group showed interstitial widening, infiltration of inflammatory cells, and hemorrhage in the lung tissue after ASCI, which gradually worsened over time, resulting in alveolar structural damage at 72 h. In comparison to the Rapa sham group, the Rapa injury group showed infiltration of inflammatory cells and hemorrhage in lung tissue after ASCI, which gradually decreased over time and were not significantly different from those in the sham group at 24 h after injury. Statistical evaluations were performed for pathological scores and the W/D at each time point (Fig. 2b and c). Compared with those in the Veh sham group, the pathological scores and W/D in lung tissue in the Veh injury group increased after ASCI at 12 h (*P* < 0.05) and continued to rise over time until 72 h (*P* < 0.05). Compared with those in the Rapa sham group, the pathological scores and W/D in the lung tissue in the Rapa injury group increased after ASCI at 12 h (*P* < 0.05), but returned to normal levels at 24 h. The differences compared with the sham group were not statistically significant. The pathological scores and W/D of the lung tissue from the Rapa injury group were lower compared with those in the Veh injury group at 24 to 72 h after injury (*P* < 0.05).

### Apoptosis of lung tissue cells following ASCI

**Figure 3:**
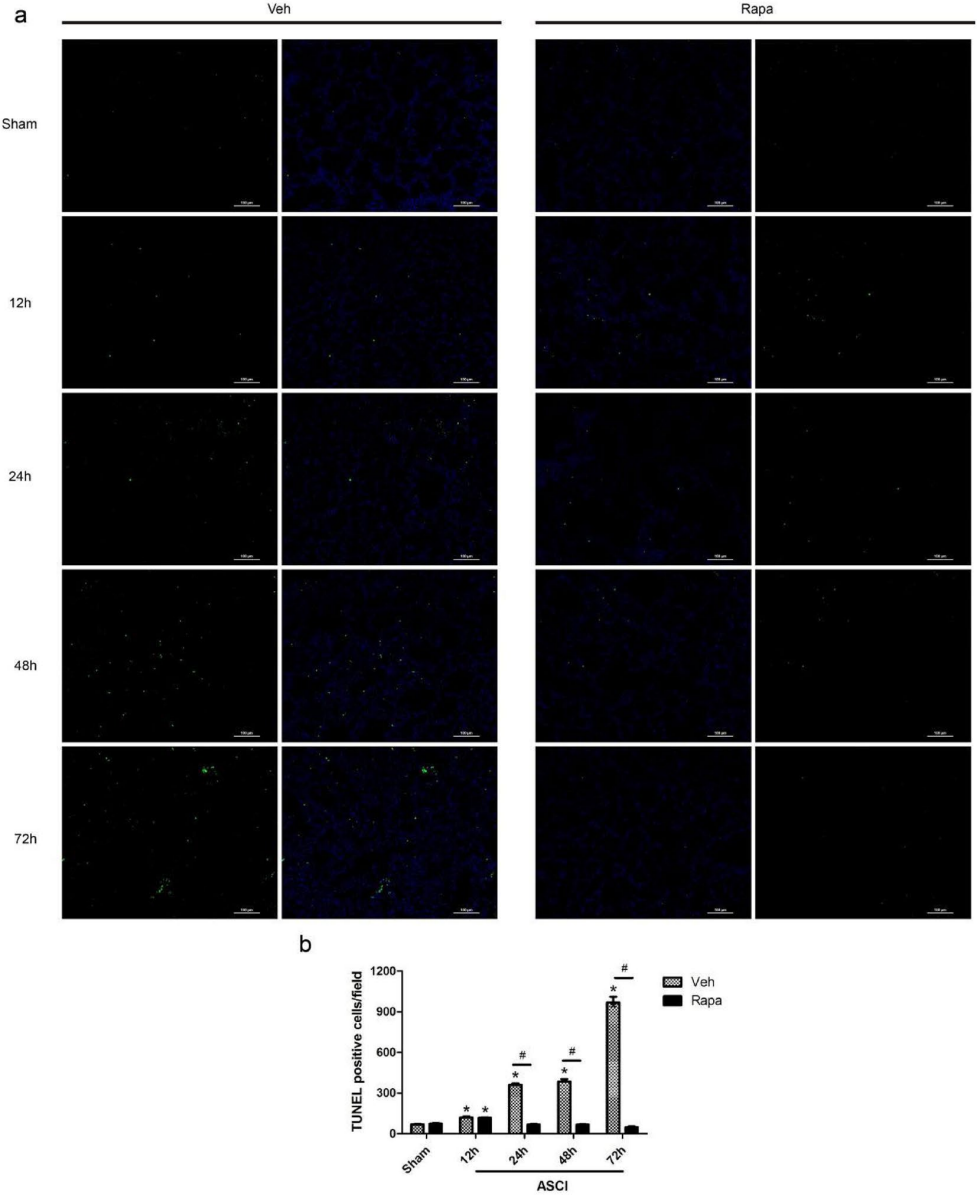
Cellular apoptosis in the lung tissue sections. (**a**) Fluorescent green dots represent TUNEL staining of nuclei containing DNA strand breaks. Representative results of lung sections at 12, 24, 48, and 72 h are shown. Magnification × 200. (**b**) Number of cells with TUNEL positivity (mean ± SD). One-way analysis of variance and the Least Significant Difference test were used to compare the data. *P < 0.05 vs. the sham group (#*p* < 0.05). Veh, vehicle; Rapa, rapamycin; ASCI, acute spinal cord injury; TUNEL, TdT-mediated dUTP-nick end labeling.

Lung cell apoptosis levels were assessed using TUNEL staining (Fig. 3a), in which the number of apoptotic cells in each field of view was counted for statistical analysis (Fig. 3b), serving as an index to investigate the lung injury caused by ASCI. At 12 h post-ASCI, the number of TUNEL-positive nuclei (containing DNA strand breaks) increased in the Veh and Rapa injury groups compared with that in their respective sham groups (Veh and Rapa), and this difference was statistically significant (*P* < 0.05). Over time, the number of apoptotic cells in the Veh injury group increased compared with that in the Veh sham group (24–72 h post-ASCI) (*P* < 0.05), and the apoptotic cell number also increased compared with that in the Rapa injury group at the same time points (24–72 h post-ASCI) (*P* < 0.05). However, the difference in the number of apoptotic cells between the Rapa injury group and the Rapa sham group was not statistically significant from 24 to 72 h post-ASCI.

### Lung autophagy post-ASCI

**Figure 4:**
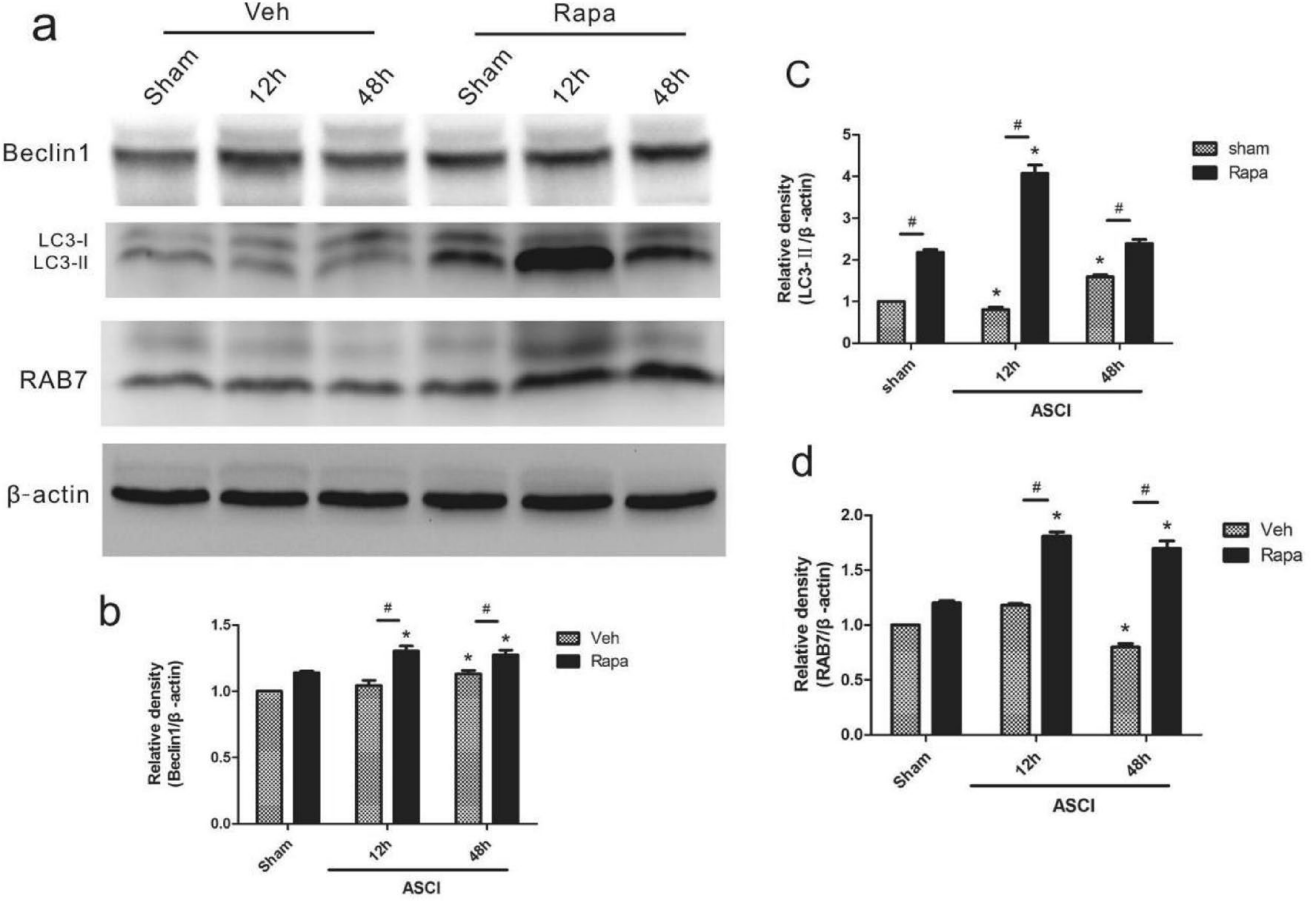
Semi-quantitative assessment of autophagy markers Beclin1, LC3, and RAB7. (**a**) Western blotting analysis of Beclin1, LC3, and RAB7 levels in lung homogenates at 12 and 48 h. The loading control was β-actin. (**b**–**d**) Beclin1, LC3, and RAB7 relative quantification from the blot shown in (a) (mean ± SD). One-way analysis of variance and the Least Significant Difference test were used to compare the data.*P < 0.05 vs. the sham group (#*p* < 0.05). Veh, vehicle; Rapa, rapamycin; ASCI, acute spinal cord injury.

LC3, Beclin1, and RAB7 are all markers of the autophagy process. We caried out semiquantitative analysis of protein levels at all time points in each group (Fig. 4a) and statistical analysis was performed (Fig. 4b–d). Compared with those in the Veh sham group, the levels of Beclin1 and RAB7 in the Veh injury group showed no significant difference art 12 h after injury, while the level of LC3 decreased (*P* < 0.05) at that time. At 48 h after injury, Beclin1 and LC3 levels increased (*P* < 0.05) and RAB7 levels decreased (*P* < 0.05). Compared with those in the Rapa sham group and the Veh injury group, the levels of Beclin1 and RAB7 in Rapa injury group increased (*P* < 0.05) after both 12 h and 48 h of injury. Compared with that in the Rapa sham group, the level of LC3 in the Rapa injury group increased (*P* < 0.05) at 12 h after injury, but showed no significant difference at 48 h. Compared with that in the Veh injury group, the level of LC3 in the Rapa injury group increased (*P* < 0.05) at 12 h and 48 h after injury (Supplementary material).

### RAB7 and LC3 colocalization

**Figure 5:**
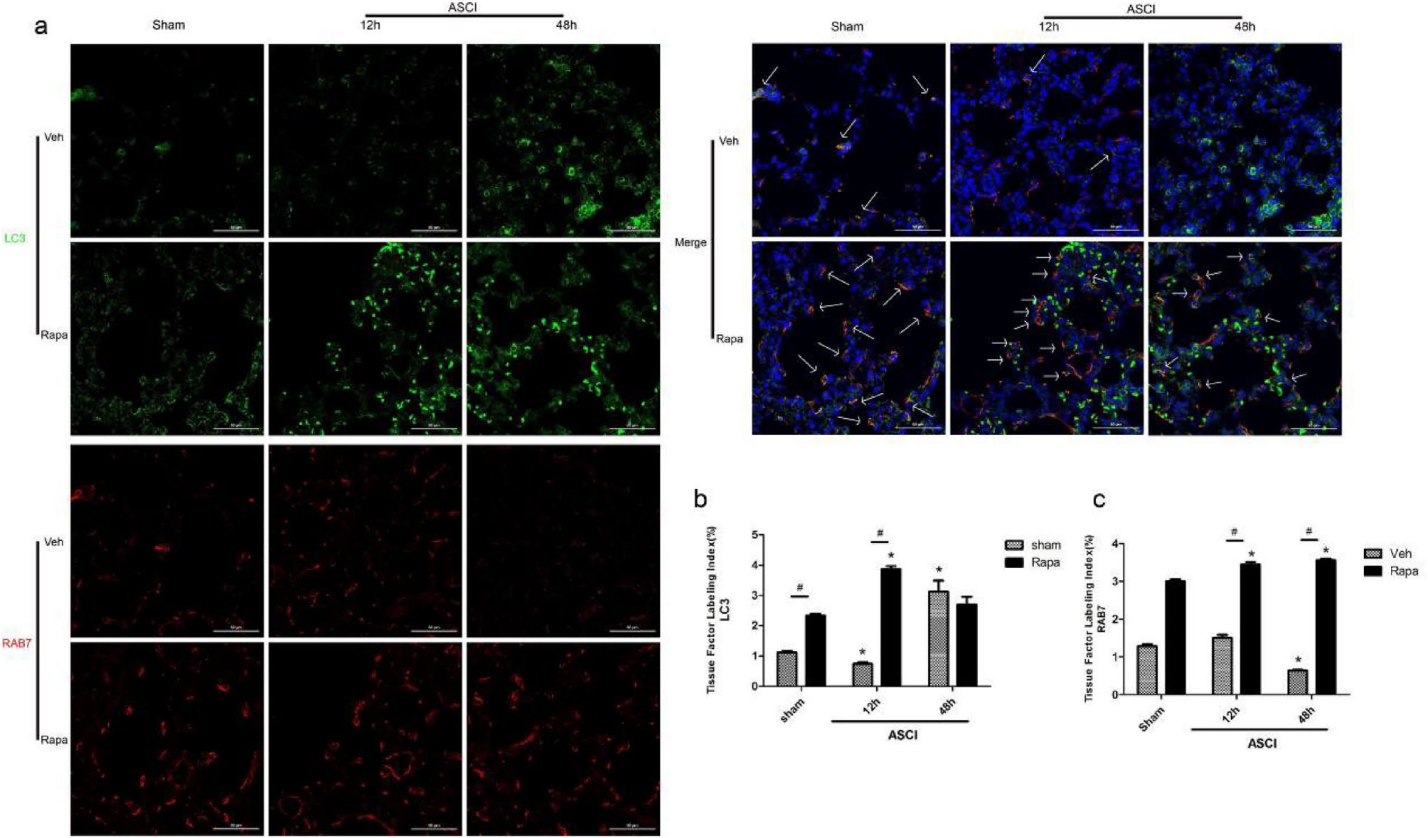
Colocalization of LC3 and RAB7. (**a**) Representative laser confocal microscopy image in which green fluorescence marks LC3 and red fluorescence marks RAB7. Magnification × 600. LC3 and RAB7colocalizatio is marked using white arrowheads. (**b**, **c**) The tissue factor labeling index for LC3 and RAB7 (mean ± SD). One-way analysis of variance and the Least Significant Difference test were used to compare the data. *P < 0.05 vs. the sham group (#*p* < 0.05). Veh, vehicle; Rapa, rapamycin; ASCI, acute spinal cord injury.

RAB7 co-localization with LC3 (an autophagosome marker) indicates autophagosomes integrating with lysosomes. Under laser confocal microscopy, LC3-II appeared as green fluorescent dots, and RAB7 showed red fluorescence (Fig. 5a), with co-localization appearing as yellow fluorescence (white arrow). The amount of co-localization fluorescence was lower in the Veh group compared with that in the Rapa group at 12 h and 48 h post injury. In the Veh group, the amount of co-localization fluorescence decreased with time after injury, while there was no significant change in the Rapa group. The statistical results (Fig. 5b and c) showed that LC3 levels in the Veh injury group decreased compared with those in the Veh sham group at 12 h post injury (*P* < 0.05), but increased at 48 h post injury (*P* < 0.05). LC3 levels in the Rapa injury group increased compared with those in the Rapa sham group at 12 h post injury (*P* < 0.05), but there was no significant difference at 48 h post injury. At 12 h post injury, LC3 levels in the Rapa group were higher than those in the Veh group (*P* < 0.05). The RAB7 levels in the Veh injury group decreased compared with those in the Veh sham group at 48 h post injury (*P* < 0.05). The RAB7 levels in the Rapa injury group increased compared with those in both the Rapa sham group and the Veh injury group at 12 h and 48 h post injury (*P* < 0.05).

### ULK-1 and AMPK expression in the lung after ASCI

**Figure 6:**
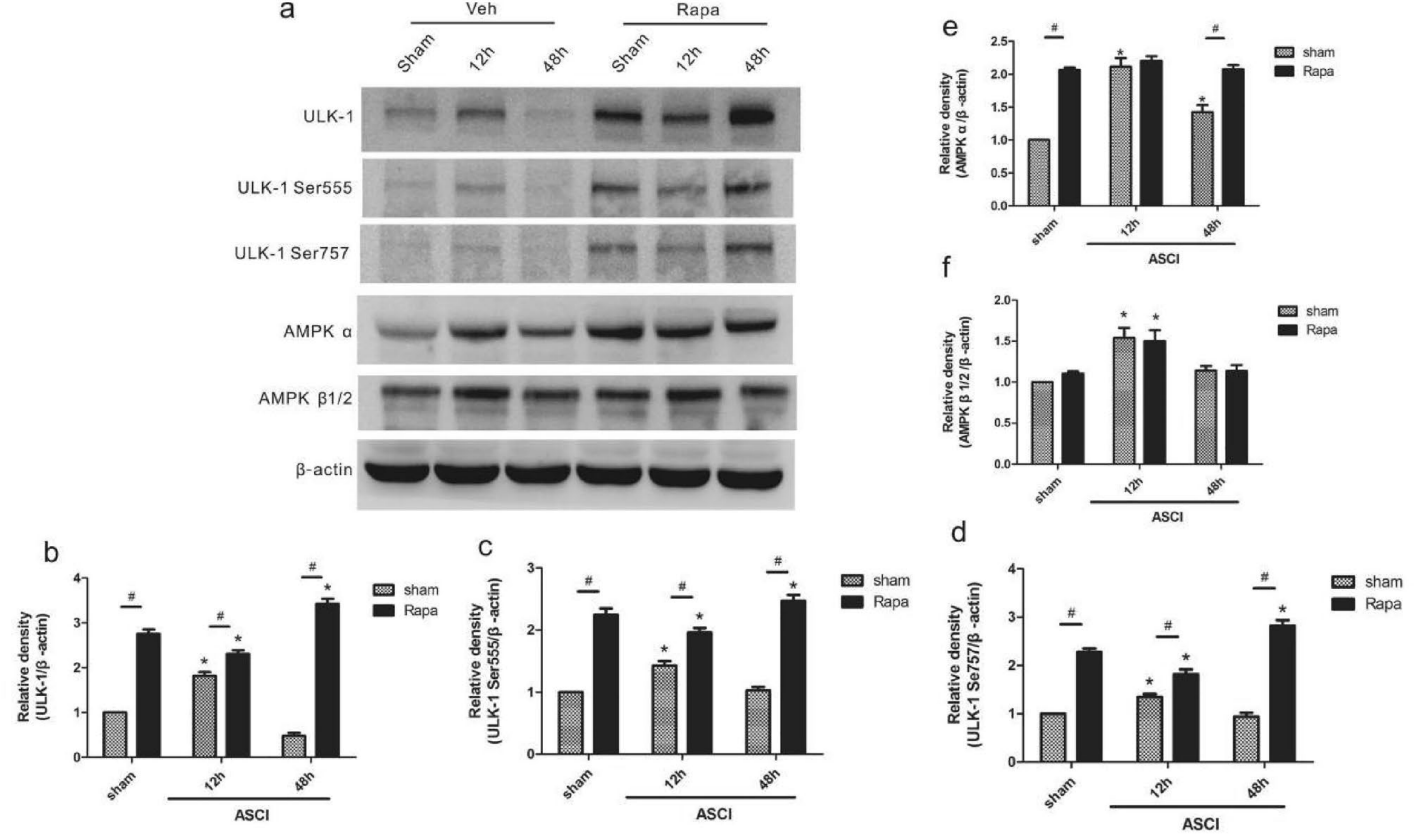
Semi-quantitative assessment of ULK-1, ULK-1 Ser555, ULK-1 Ser757, AMPKα, and AMPKβ1/2. (**a**) Western blotting analysis of ULK-1, ULK-1 Ser555, ULK-1 Ser757, AMPKα, and AMPKβ1/2levels in lung homogenates at 12 and 48 h. The loading control was β-actin. (**b**–**f**) Relative levels of ULK-1, ULK-1 Ser555, ULK-1 Ser757, AMPKα, and AMPKβ1/2 derived from the blot shown in (a) (mean ± SD). One-way analysis of variance and the Least Significant Difference test were used to compare the data.*P < 0.05 vs. the sham group (#*p* < 0.05).

To investigate the possible mechanisms of the observed changes in lung tissues after ASCI, we analyzed the levels of ULK-1, ULK-1 Ser555, ULK-1 Ser757, AMPKα, and AMPKβ1/2 using western blotting analysis (Fig. 6a) and performed statistical analysis (Fig. 6b–f). After Rapa pretreatment, the levels of ULK-1, ULK-1 Ser555, and ULK-1 Ser757 were higher in the Sham state at 12 and 48 h after injury compared with those in the Veh group. In the Rapa injury group, the levels of ULK-1, ULK-1 Ser555, and ULK-1 Ser757 decreased at 12 h (*P* < 0.05) and increased at 48 h (*P* < 0.05) compared with those in the Rapa sham group. In the Veh injury group, the levels of ULK-1, ULK-1 Ser555, and ULK-1 Ser757 increased at 12 h (*P* < 0.05) and showed no statistically significant difference at 48 h compared with those in the Veh sham group. The levels of AMPKβ1/2 in the Rapa injury group and Veh injury group increased at 12 h (*P* < 0.05) compared with those in the Rapa sham group and Veh sham group, respectively. The levels of AMPKα in the Rapa injury group showed no statistically significant difference compared with those in the Rapa sham group. AMPKα levels in the Veh injury group increased at 12 h and 48 h (*P* < 0.05) compared with those in the Veh sham group (Supplementary material).

## Discussion

Bao et al.^8^ showed that compared with other patients with severe multiple injuries, the degree of systemic inflammatory response in patients with ASCI was more severe. Moreover, the severity of the injury does not change with the location of the injured spinal cord. Researchers believe that such differences might be related to the specificity of central nervous system injury; however, the specific mechanism is still unclear. Sun et al.^9^ found that the use of veratrol can reduce lung injury after ASCI, but did not find a clear mechanism. Post-ASCI lung injury greatly threatens the life of patients and plays an important role in the occurrence of death. Therefore, it is important to clarify its possible mechanism to reduce the mortality of patients with ASCI.

The mammalian target of rapamycin (mTOR) inhibitor, Rapamycin, was first used in clinical practice as a potent maintenance immunosuppressant for the treatment of transplant rejection^10^. Meanwhile, rapamycin can activate autophagy by specifically inhibiting mTOR. Autophagy is a lysosome-dependent degradation pathway that is unique to eukaryotic cells and is important for maintaining their homeostasis^11–13^. Autophagy as a novel cell regulated death pathway provides a new perspective for the current study of lung injury induced by different factors^14^.

Wenlin et al.^15^ demonstrated that rapamycin could alleviate paraquat-induced pulmonary fibrosis through the NFE2 like BZIP transcription factor 2 (Nrf2) pathway. However, the methods by which different factors induce lung injury in animal models are not exactly the same, and rapamycin does not always exert a protective function. Research on lipopolysaccharide (LPS)-induced acute lung injury showed that mTOR was activated after LPS challenge, and this state led to increased IL1β and IL18 in lung tissue. Rapamycin can protect lung tissue by inhibiting the production of IL1β and IL18 by inhibiting mTOR and activating autophagy^16^. The activation of autophagy can remove damaged mitochondria and prevent the release of mitochondrial reactive oxygen species, thereby inhibiting the secretion of IL1β and IL18^17,18^. However, a study by Jill et al.^19^ found that rapamycin aggravated the extent of LPS-induced lung injury through signal transducer and activator of transcription 1 (STAT1)-mediated associated apoptosis. Jia et al.^16^ believed that the difference effects of rapamycin in LPS-induced lung injury might be related to the LPS dose and the dose and administration time of rapamycin. A study by Heng et al.^20^ found that the expression of autophagy biomarkers, such as Beclin1 and LC3, in lung tissue increased after limb ischemia/reperfusion, and autophagy was activated. Limb ischemia/reperfusion-induced lung injury was attenuated after autophagy inhibition using Tetrahydropalmatine (THP) and 3-MA. However, Fan et al.^21^ found that rapamycin could regulate the release of inflammatory mediators by activating autophagy, thereby reducing lung damage caused by limb ischemia/reperfusion by decreasing the damage done to alveolar epithelial cells. Current findings illustrate that the role of autophagy differs under different injury factors; therefore, the effect and mechanism after rapamycin intervention is completely different.

Our results suggested that the discrepancy in these findings might be because of the short duration of observation of autophagy in lung injury. The longest observation time in the above studies was 12 h post damage, and the observation time points were different in studies by different researchers. This is because these disease models had a short duration of lung injury. In this case, a short duration of lung injury is not useful in our study of the role of autophagy. Our previous research results showed that the damage to the lung after ASCI lasted until 72 h after ASCI, and that lung autophagy post-ASCI was in a dynamic state. After ASCI, the formation of early autophagosomes in the lungs decreased and late autophagosomes accumulated excessively, and autophagy was always inhibited during this change process. Therefore, this study observed the early (12 h) and late (48 h) periods of lung tissue damage after ASCI. The selection of these time points is conducive to our more objective and comprehensive understanding of the role and possible mechanism of rapamycin in post-ASCI lung injury. We found that at 12 h post-ASCI, compared with those in the sham operation group, the Rapa injury group and the Veh injury group had scattered hemorrhage points in the lungs, mild interstitial enlargement, mild inflammatory cell infiltration, and mild pulmonary edema. Alveolar structure was normal at 12 h after ASCI. As time went by (48 h after injury), the lung injury, hemorrhage, interstitial widening, inflammation, and edema in the Rapa injury group improved significantly, and there was no significant difference compared with the sham operation group. However, the damage of the lungs in the Veh injury group, such as hemorrhage, interstitial widening, inflammation, and edema, were significantly aggravated compared with those in the sham operation group. At 72 h after ASCI, the alveoli of the Veh injury group collapsed without a normal alveolar structure. This shows that rapamycin can reduce the degree of pathological damage to the lung after ASCI, including pulmonary edema and pulmonary hemorrhage and other damage. Wang et al.^22^ showed that the secretion of inflammatory factors (IL-1β, TNF-α) and the degree of inflammatory response of rapamycin-treated macrophages were reduced after stimulation using cecal contents. In vivo studies have shown that rapamycin has a protective effect on sepsis during acute infection, attenuates the pathological damage of affected organs, and its pretreatment can significantly reduce the mortality of animals with cecal ligation and puncture (CLP) sepsis. In addition, that study found that postoperative administration of rapamycin was more beneficial for CLP sepsis, which also confirmed the clinical value of rapamycin treatment. Our experimental results also showed that there were significantly fewer dead cells in the lungs of the Rapa injury group compared with those in the Veh injury group. This result supports the clinical use of rapamycin to prevent and treat ASCI-induced lung injury.

Our study found that the levels of Beclin1, RAB7, and LC3 in the Rapa injury group were significantly increased after ASCI compared with those in the Veh injury group. This autophagy activation lasted until 48 h after injury, and the levels of Beclin1, RAB7, and LC3 remained high. Thus, rapamycin activated lung autophagy after ASCI and attenuated the degree of lung injury. Wei et al.^23^ showed that autophagy was enhanced by rapamycin: In mitochondrial damage-associated molecular patterns-induced lung injury, inflammatory cytokine secretion, lung injury extent, and NLRP3 inflammasome-associated protein levels were markedly reduced. Activation of autophagy alleviates lung injury^23^. This is consistent the present findings. Our study found that the Rapa injury group still had slight lung injury at 12 h after ASCI and was not completely relieved. We hypothesized that the mechanism of this phenomenon might be related to AMPK-mTORC-ULK1 regulatory axis.

Our results demonstrated a significant increase in AMPK levels in the Rapa injury group compared with those in the Veh injury group, among which AMPKα was the most significant. In the Veh injury group, AMPKα levels were increased significantly at 12 h after ASCI, and remained higher compared with that in the Veh sham group at 48 h after ASCI. Although MPKα levels in the Rapa group were significantly higher compared with those in the Veh group (except at 12 h after injury), there was no significant difference between the Rapa injury group compared with the sham group at the various time points. The level of AMPKα in the Rapa group did not change after injury. On the contrary, the Veh group showed obvious fluctuations after the injury (significantly increase in the early stage and a slight increase in the late stage). AMPK is an important requirement for epithelial and endothelial integrity, and has a protective role in organ damage after acute ischemic events^24^. Activation of AMPKα can enhance the repair capability of capillary endothelial cells and promote the repair of microvessels in the lung, thereby reducing tissue permeability and injury^25^.

ULK-1 Ser757 is the binding site between mTOR and ULK-1, and ULK-1 Ser555 is the binding site between AMPK and ULK-1. The changes in the levels of ULK-1, ULK-1 Ser757, and ULK-1 Ser555 in Rapa group and Veh group are also interesting. In the Veh group, ULK-1, ULK-1 Ser757, and ULK-1 Ser555 levels increased at 12 h after injury compared with those in the sham group and then returned to baseline levels at 48 h after injury. In the Rapa group, ULK-1, ULK-1 Ser757, and ULK-1 Ser555 levels were significantly higher than those in the Veh group after sham operation, and at 12 h and 48 h after injury; however, the changes of their levels after injury were different between the Veh group and the Rapa group. There was a transient increase in the Veh group, but a transient decrease in the Rapa group. This difference appeared at 12 h after injury. Interestingly, at this time point (12 h after injury), the level of AMPKα in the Rapa injury group did not change significantly, but increased in the Veh injury group. Our study found that the Rapa group still had slight lung damage at 12 h after ASCI. Consequently, we inferred that the possible reason is that the level of AMPKα in the Rapa group did not change significantly, and rapamycin, as an mTORC inhibitor, had no clear and direct effect on the expression of AMPK. The key to this regulatory mechanism might be related to the AMPK-mTORC-ULK1 regulatory axis. Autophagy is tightly controlled by two sensor elements: mTORC and AMPK. mTORC is a master regulator of protein stability, while AMPK supports cellular energy homeostasis^26^. AMPK can activate autophagy by activating ULK1 and Beclin1^27^. mTORC downregulates autophagy through ULK1 under nutrient-rich conditions. There is a double negative feedback loop between mTORC and AMPK. Rapamycin activates autophagy by inhibiting mTORC, which mostly occurs under energy-sufficient conditions. Cellular stress resulting from trauma is both short-lived and massive. AMPK exerts tight control over ATP-consuming processes, including glycogen, protein, fatty acid, and cholesterol synthesis, and maintains cellular homeostasis by sensing energy levels, thereby increasing ATP production^28–30^. Activation of AMPK after ASCI can meet the extra energy demand of the body in the traumatized state through a variety of metabolic pathways. Our study showed that lung injury after ASCI can be alleviated via activation of autophagy, and rapamycin can effectively promote this protective process. However, there was still slight lung injury in the early stage (12 h) after rapamycin intervention, which was related to the blockage of autophagic flow. This blockage might be related to a lack of AMPK activation after trauma, resulting in a relatively insufficient energy supply, which cannot meet the needs of autophagy. This lack of energy would hinders autophagic flux, and we believe that the key to the regulation of this process is the balance of the AMPK-mTORC-ULK1 regulatory axis. Szymanska et al.^31^ developed a mathematical model to clarify the interactions among AMPK, mTORC1 and ULK1, which focused on inducing autophagy via various stressors. Although Szymanska et al.^32^ proposed a mechanism for the AMPK-mTORC1-ULK1 regulatory network, they did not consider many unstudied cellular stress events, such as those occurring in the lung after ASCI. Their mechanistic model cannot be used to evaluate, predict, and explain all cellular stress events. Understanding the precise molecular balance among AMPK, mTORC1, andULK1, especially the regulation of autophagy, has important implications to regulate cell survival and some cell stress-related diseases, and will help to promote advanced treatments for these diseases.

## Conclusions

Our study found that after rapamycin pretreatment, the pathological damage of the lung after ASCI, such as pulmonary hemorrhage, pulmonary edema, and inflammatory infiltration, was alleviated. This therapeutic effect might be associated with lung autophagy activation by rapamycin through the AMPK-mTORC1-ULK1 regulatory axis. Our results provide a new perspective and theoretical basis for effectively preventing the development of post-ASCI lung injury, and its treatment using rapamycin. In this severe traumatic disease, further research on the AMPK-mTORC1-ULK1 regulatory axis is required, and it might be recognized an important new mechanism for regulating the cell state and maintaining organ function.

## Supplementary Information

Supplementary Figures.

## Data Availability

All data generated or analysed during this study are included in this published article.

## References

[1] Adegeest CY (2022). Influence of severity and level of injury on the occurrence of complications during the subacute and chronic stage of traumatic spinal cord injury: A systematic review. J. Neurosurg. Spine.

[2] DeVivo MJ, Chen Y, Wen H (2022). Cause of death trends among persons with spinal cord injury in the United States: 1960–2017. Arch. Phys. Med. Rehabil..

[3] Urdaneta F, Layon AJ (2003). Respiratory complications in patients with traumatic cervical spine injuries: Case report and review of the literature. J. Clin. Anesth..

[4] He B, Nan G (2016). Pulmonary edema and hemorrhage after acute spinal cord injury in rats. Spine J..

[5] Anandasivam NS, Ondeck NT, Bagi PS, Galivanche AR, Samuel AM, Bohl DD, Grauer JN (2021). Spinal fractures and/or spinal cord injuries are associated with orthopedic and internal organ injuries in proximity to the spinal injury. N. Am. Spine Soc. J..

[6] Chu R, Wang J, Bi Y, Nan G (2018). The kinetics of autophagy in the lung following acute spinal cord injury in rats. Spine J..

[7] Qin L (2020). Mechanistic target of rapamycin-mediated autophagy is involved in the alleviation of lipopolysaccharide-induced acute lung injury in rats. Int. Immunopharmacol..

[8] Bao F, Bailey CS, Gurr KR, Bailey SI, Rosas-Arellano MP, Dekaban GA, Weaver LC (2009). Increased oxidative activity in human blood neutrophils and monocytes after spinal cord injury. Exp. Neurol..

[9] Liu J, Yi L, Xiang Z, Zhong J, Zhang H, Sun T (2015). Resveratrol attenuates spinal cord injury-induced inflammatory damage in rat lungs. Int. J. Clin. Exp. Pathol..

[10] Webster AC, Lee VW, Chapman JR, Craig JC (2006). Target of rapamycin inhibitors (sirolimus and everolimus) for primary immunosuppression of kidney transplant recipients: a systematic review and meta-analysis of randomized trials. Transplantation.

[11] Mizushima N, Komatsu M (2011). Autophagy: Renovation of cells and tissues. Cell.

[12] Majcher V, Goode A, James V, Layfield R (2015). Autophagy receptor defects and ALS-FTLD. Mol. Cell Neurosci..

[13] Reggiori F, Klionsky DJ (2002). Autophagy in the eukaryotic cell. Eukaryot. Cell.

[14] Tang PS, Mura M, Seth R, Liu M (2008). Acute lung injury and cell death: How many ways can cells die. Am. J. Physiol. Lung Cell. Mol. Physiol..

[15] Tai W (2020). Rapamycin attenuates the paraquat-induced pulmonary fibrosis through activating Nrf2 pathway. J. Cell Physiol..

[16] Jia X, Cao B, An Y, Zhang X, Wang C (2019). Rapamycin ameliorates lipopolysaccharide-induced acute lung injury by inhibiting IL-1β and IL-18 production. Int. Immunopharmacol..

[17] Nakahira K (2011). Autophagy proteins regulate innate immune responses by inhibiting the release of mitochondrial DNA mediated by the NALP3 inflammasome. Nat. Immunol..

[18] Chuang SY, Yang CH, Chou CC, Chiang YP, Chuang TH, Hsu LC (2013). TLR-induced PAI-2 expression suppresses IL-1β processing via increasing autophagy and NLRP3 degradation. Proc. Natl. Acad. Sci. U.S.A..

[19] Fielhaber JA (2012). Inhibition of mammalian target of rapamycin augments lipopolysaccharide-induced lung injury and apoptosis. J. Immunol..

[20] Wen H, Zhang H, Wang W, Li Y (2020). Tetrahydropalmatine protects against acute lung injury induced by limb ischemia/reperfusion through restoring PI3K/AKT/mTOR-mediated autophagy in rats. Pulm. Pharmacol. Ther..

[21] Fan T (2020). Autophagy decreases alveolar epithelial cell injury by regulating the release of inflammatory mediators. J. Cell. Physiol..

[22] Wang Z, Li Y, Yang X, Zhang L, Shen H, Xu W, Yuan C (2019). Protective effects of rapamycin induced autophagy on CLP septic mice. Comp. Immunol. Microbiol. Infect. Dis..

[23] Peng W (2021). Autophagy alleviates mitochondrial DAMP-induced acute lung injury by inhibiting NLRP3 inflammasome. Life Sci..

[24] Hayes HV, Wolfe V, O'Connor M, Levinsky NC, Piraino G, Zingarelli B (2021). Deficiency of AMPKα1 exacerbates intestinal injury and remote acute lung injury in mesenteric ischemia and reperfusion in mice. Int. J. Mol. Sci..

[25] Jian MY, Alexeyev MF, Wolkowicz PE, Zmijewski JW, Creighton JR (2013). Metformin-stimulated AMPK-α1 promotes microvascular repair in acute lung injury. Am. J. Physiol. Lung Cell. Mol. Physiol..

[26] Holczer M, Hajdú B, Lőrincz T, Szarka A, Bánhegyi G, Kapuy O (2019). A double negative feedback loop between mTORC1 and AMPK kinases guarantees precise autophagy induction upon cellular stress. Int. J. Mol. Sci..

[27] Tamargo-Gómez I, Mariño G (2018). AMPK: Regulation of metabolic dynamics in the context of autophagy. Int. J. Mol. Sci..

[28] Shaw RJ, Kosmatka M, Bardeesy N, Hurley RL, Witters LA, DePinho RA, Cantley LC (2004). The tumor suppressor LKB1 kinase directly activates AMP-activated kinase and regulates apoptosis in response to energy stress. Proc. Natl. Acad. Sci. U.S.A..

[29] Hardie DG (2007). AMP-activated/SNF1 protein kinases: Conserved guardians of cellular energy. Nat. Rev. Mol. Cell. Biol..

[30] Alers S, Löffler AS, Wesselborg S, Stork B (2012). Role of AMPK-mTOR-Ulk1/2 in the regulation of autophagy: Cross talk, shortcuts, and feedbacks. Mol. Cell. Biol..

[31] Szymańska P, Martin KR, MacKeigan JP, Hlavacek WS, Lipniacki T (2015). Computational analysis of an autophagy/translation switch based on mutual inhibition of MTORC1 and ULK1. PLoS ONE.

[32] Kensler TW, Wakabayashi N, Biswal S (2007). Cell survival responses to environmental stresses via the Keap1-Nrf2-ARE pathway. Annu. Rev. Pharmacol. Toxicol..

