# Supplementary material for: Rapamycin prevents lung injury related to acute spinal cord injury in rats
Source: Sci Rep. 2023 Jul 1;13:10674. doi: 10.1038/s41598-023-37884-6 (PMC10314925; doi:10.1038/s41598-023-37884-6)
Supplement: Supplementary file 1 — Supplementary Figures. [file 41598_2023_37884_MOESM1_ESM.pdf]

a b c

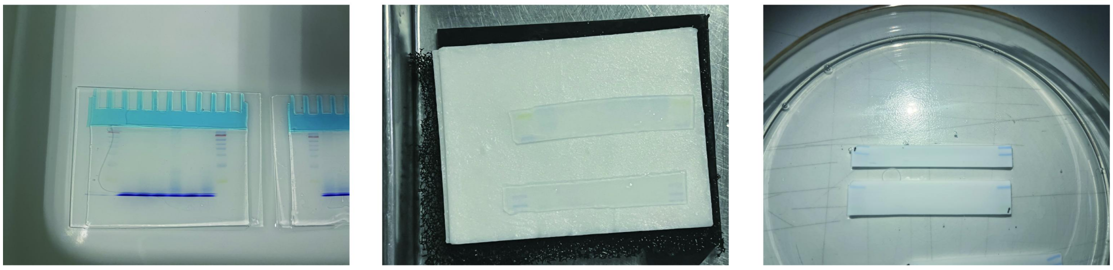

Sfig1 Western Blotting Analysis **(a)** The resulting total proteins were mixed with 5 x SDS-PAGE loading buffer and subjected to electrophoresis on Tris-glycine gels. Edge lanes are marked with colored marker proteins to represent different molecular weights. (Thermo Fisher Scientific, Waltham, MA, USA) **(b)** According to the molecular weight of the target protein, the reference cutting gel is based on the marker protein to ensure a sufficient molecular weight range. **(c)** The separated proteins were transferred electrophoretically onto polyvinylidene fluoride membranes.

a b c d

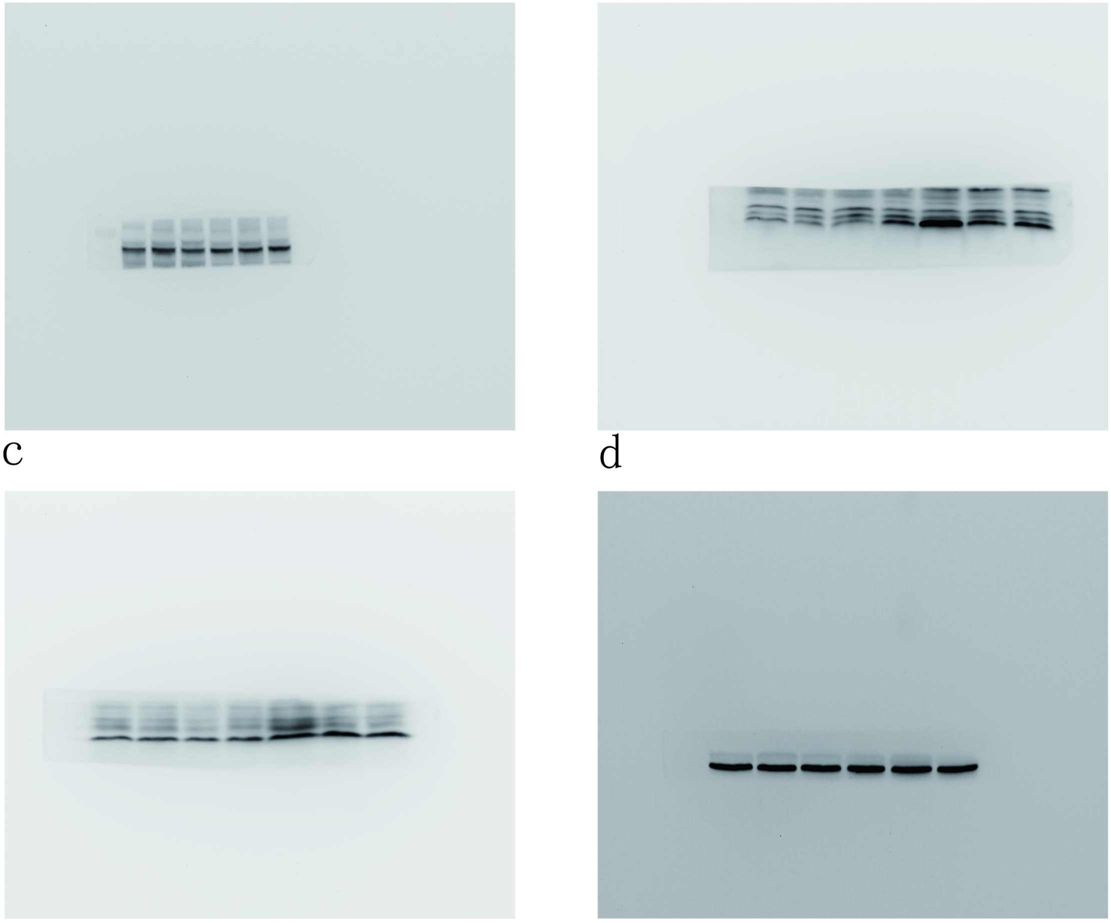

Sfig2 Original data of Western Blotting **(a)** Beclin1 **(b)** LC3 **(c)** RAB7 **(d)** β-actin

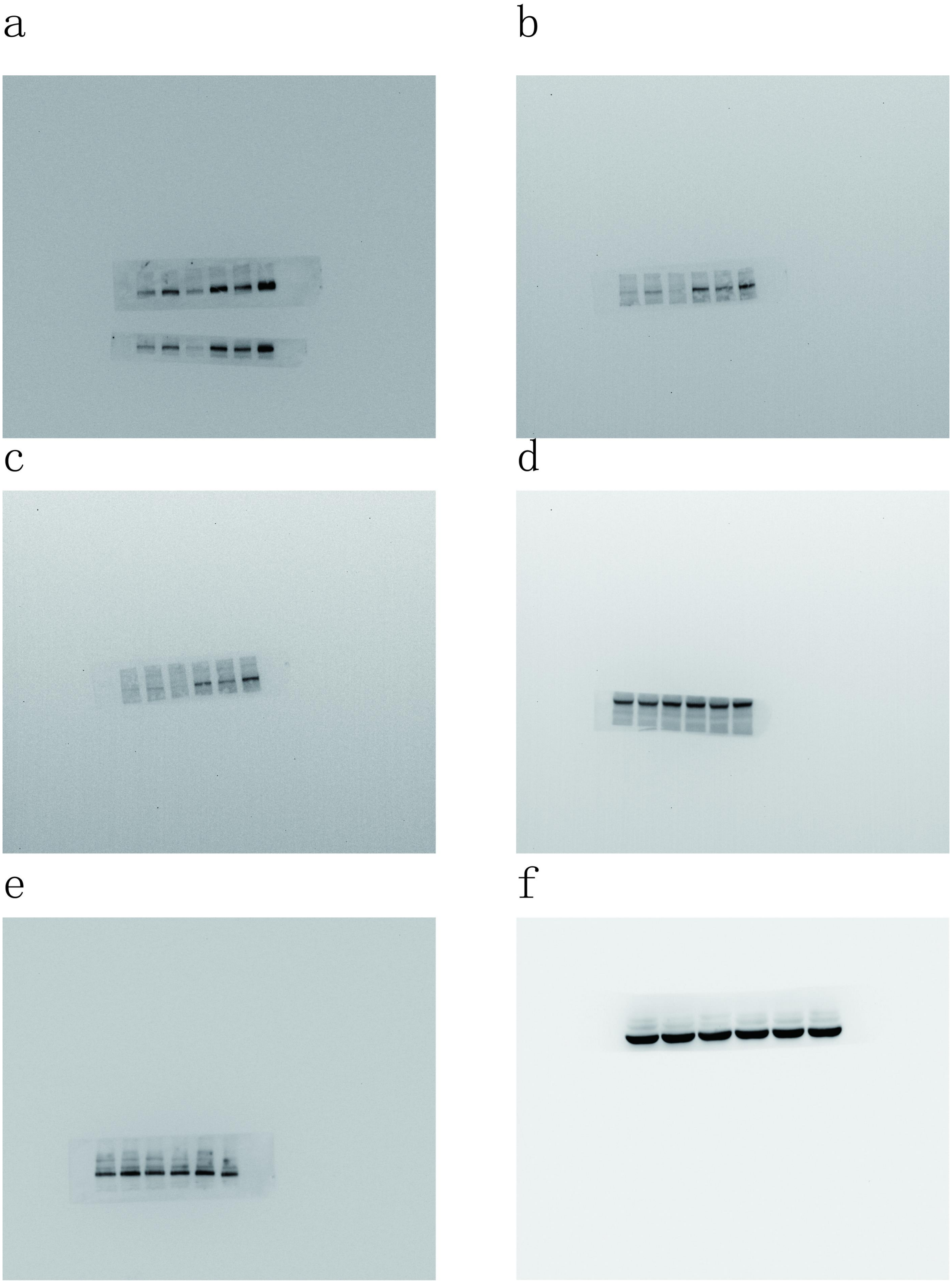

Sfig3 Original data of Western Blotting **(a)** ULK-1 **(b)** ULK-1 Ser555 **(c)** ULK-1 Ser757 **(d)** AMPK-α **(e)** AMPK-β **(f)** β-actin
